# Regulation of HIF-1α and VEGF by miR-20b Tunes Tumor Cells to Adapt to the Alteration of Oxygen Concentration

**DOI:** 10.1371/journal.pone.0007629

**Published:** 2009-10-29

**Authors:** Zhang Lei, Bo Li, Zhuoshun Yang, Haoshu Fang, Gui-Mei Zhang, Zuo-Hua Feng, Bo Huang

**Affiliations:** Department of Biochemistry & Molecular Biology, Tongji Medical College, Huazhong University of Science & Technology, Wuhan, The People's Republic of China; Texas A&M University, United States of America

## Abstract

The regulation of HIF-1α is considered to be realized by pVHL-mediated ubiquitin-26S proteasome pathway at a post-transcriptional level. The discovery of a class of small noncoding RNAs, called microRNAs, implies alternative mechanism of regulation of HIF-1α. Here, we show that miR-20b plays an important role in fine-tuning the adaptation of tumor cells to oxygen concentration. The inhibition of miR-20b increased the protein levels of HIF-1α and VEGF in normoxic tumor cells; the increase of miR-20b in hypoxic tumor cells, nevertheless, decreased the protein levels of HIF-1α and VEGF. By using luciferase reporter vector system, we confirmed that miR-20b directly targeted the 3′UTR of *Hif1a* and *Vegfa*. On the other hand, the forced overexpression of HIF-1α in normoxic tumor cells downregulated miR-20b expression. However, HIF-1α knockdown in hypoxic tumor cells caused the increase of miR-20b. The differential expression of miR-20b has important biological significance in tumor cells, either enhancing the growth or favoring the survival of tumor cells upon the oxygen supply. Thus, we identify a novel molecular regulation mechanism through which miR-20b regulates HIF-1α and VEGF and is regulated by HIF-1α so to keep tumor cells adapting to different oxygen concentrations.

## Introduction

Hypoxia is a common feature in solid tumors as the consequence of poor tumor vascularization [1]–[3]. The transcription factor hypoxia-inducible factor-1 (HIF-1) is a key regulator responsible for the induction of genes that facilitate adaptation and survival of tumor cells from hypoxic microenvironment and confer the tumor a worse malignant phenotype [4], [5]. As a heterodimeric complex, HIF-1 consists of a hypoxically inducible subunit HIF-1α and a constitutively expressed subunit HIF-1β. The overexpression of HIF-1α was found in various types of cancers of both human and mouse [4], [6]. To date, the regulation of HIF-1α by oxygen is elucidated well. Under normoxia, hydroxylation of two proline residues and acetylation of a lysine residue of HIF-1α are mediated by oxygen. Such modifications cause tumor suppressor von Hippel-Lindau protein (pVHL) to bind and degrade HIF-1α through ubiquitin-26S proteasome system. Nevertheless, in hypoxia, the hydroxylation is inhibited by the lack of oxygen, leading to no pVHL binding and the stability of HIF-1α [4]–[6]. During tumorigenesis, the hypoxic microenvironment and/or genetic alteration pVHL may cause a high level of HIF-1α in cancer cells [7], [8], suggesting that HIF-1α is a potential target in tumor therapy.

The regulation of HIF-1α must be tight in cells in order to precisely adapt to changes of oxygen supply. In this regard, the mechanisms of regulating HIF-1α might be delicate and complex. Although pVHL-mediated degradation of HIF-1α is an important pathway, phosphorylation of HIF-1α also plays a role by increasing the transcriptional activity of HIF-1α [9], [10]. Moreover, cytokines, growth factors, and environmental stimuli seem to be involved in the regulation of HIF-1α under nonhypoxic condition [11], [12]. Besides those, whether other pathway(s) involves the regulation of HIF-1α remains unclear. Recently, the intense studies on a class of small noncoding RNAs, called microRNAs (miRNAs), disclose the regulation of gene expression by miRNAs. The underlying mechanism involves miRNAs annealing to inexactly complementary sequences in the 3′-UTR of target mRNAs to suppress translation [13], [14]. In this regard, HIF-1α is possibly regulated by miRNAs. Recently, HIF-1α was reported as the target of miR-17-92 microRNA cluster in lung cancer cells [15]. In the present study, we further show a molecular mechanism involving miR-20b regulating HIF-1α and VEGF and being regulated by HIF-1α, through which tumor cells adapt to different oxygen concentrations.

## Results

### Inverse level of miR-20b and *HIF-1α* in tumor cells

**Figure 1:**
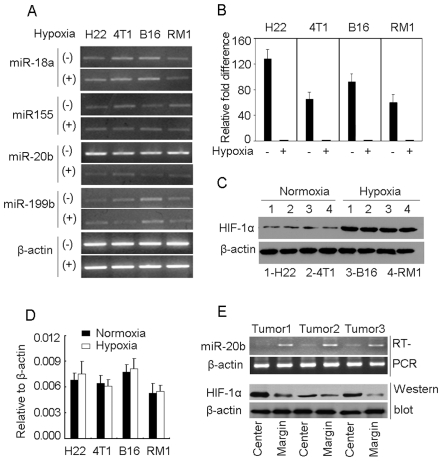
Expression of miR-20b is reversely correlated with HIF-1α level in tumor cells. (A) The expressions of miR-18a, 155, 20b and 199b in tumor cell lines were detected by RT-PCR in normoxia (20% oxygen) or hypoxia (1% oxygen). (B) The detection of miR-20b in tumor cell lines by real time RT-PCR. The miR-20b level in the hypoxia groups was designated as 1. (C and D) The expression of HIF-1α in tumor cell lines was detected by Western blot (C) or real time RT-PCR (D). (E) Expressions of miR-20b and HIF-1α in different tumor regions. 1×10^5^ H22 tumor cells were inoculated subcutaneously into mice. When tumor size was >9×9 mm, the central and marginal regions of tumor tissues were used for miR-20b and HIF-1α detection by RT-PCR and Western blot, respectively. Data from three tumor tissues were presented in this figure.

We predicted the candidate mouse microRNAs of targeting *Hif1a* by combinatorial utilization of three different algorithms, including TargetScan (http://www.targetscan.org/), PicTar (http://pictar.bio.nyu.edu/), and Sanger microRNA target (http://microrna.sanger.ac. uk/). On the basis of the obtained information, we focused our attention on miR-18a, miR-199b, miR-20b and miR-155. Four murine tumor cell lines from different tissues, including liver cancer H22, breast cancer 4T1, prostate cancer RM1 and melanoma B16, were tested here. In normoxia, miR-18a, miR-199b, miR-20b and miR-155 were expressed in such tumor cell lines with different expression levels (Figure 1A). However, compared to the normoxic condition, the expression of miR-20b, rather than miR-18a, miR-199b and miR-155, in hypoxia was strikingly decreased (Figure 1A). We also confirmed such expression pattern of miR-18a, miR-199b and miR-155 by quantitative RT-PCR (Supporting information, Figure S1). In addition, we determined miR-20a and miR-106-363 cluster other members (miR-106a, miR-18b, miR-92-2, and miR-363), since miR-20a is similar to miR-20b and miR-20b belongs to miR-106-363 cluster [16]. However, the expressions of those genes seemed not to be associated with the change of oxygen concentration (Suupporting information, Figure S2). We then focused our attention on miR-20b. The analysis by quantitative RT-PCR showed that miR-20b had 64-fold decrease in hypoxia (Figure 1B). Nevertheless, inverse to the expression pattern of miR-20b, the protein level of HIF-1α was very low in normoxia, but very high in hypoxia (Figure 1C). In addition, hypoxia seemed not to change HIF-1α mRNA expression of tumor cells, evaluated by real time RT-PCR (Figure 1D). These data suggested a relationship between miR-20b and HIF-1α expression.

Among the tested tumor cell lines, H22 cells expressed the most difference of miR-20b between normoxia and hypoxia. We therefore validated the above *in vitro* data *in vivo* by inoculating H22 cells to BALB/c mice subcutaneously. When tumors reached to the size of 9×9 mm, we tested the expression of miR-20b in different tumor regions. We found that miR-20b was much lower in the hypoxic central region and much higher in the normoxic marginal region (Figure 1E). In parallel, HIF-1α detection showed much higher in the center and much lower in the margin (Figure 1E). The low expression of miR-20b in hypoxia was not due to the tumor necrosis. Most tumor cells in the detected tumor mass were not stained by trypan blue and the mRNA levels of either β-actin or GAPDH between central region and marginal region had no significant difference, evaluated by real time RT-PCR (data not shown). Furthermore, we performed the immunohistochemical assay. As expected, the result showed that the central tumor tissues were positively stained with anti-HIF-1α antibody; however, the marginal tumor tissues were stained negatively (Supporting information, Figure S3). Thus, the expression miR-20b is inversely relative to the level of HIF-1α protein in tumor cells, implying that tumor cell HIF-1α might be a target of miR-20b.

### *HIF-1α* is targeted by miR-20b

**Figure 2:**
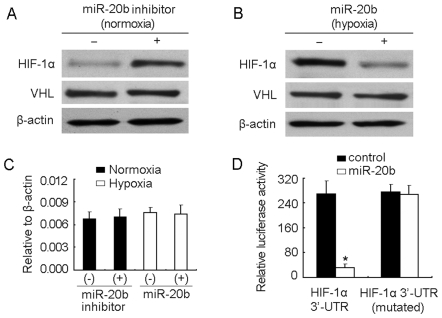
HIF-1α is targeted by miR-20b. (A) miR-20b inhibitor increased HIF-1α protein in normoxic H22 cells. miR-20b inhibitor or control oligonucleotide was transfected into normoxic H22 cells. 48 hr later, the cells were used to detected HIF-1α and VHL proteins by Western blot. (B) miR-20b decreased HIF-1α protein in hypoxic H22 cells. miR-20b or control oligonucleotide was transfected into hypoxic H22 cells. 48 hr later, the cells were used to detect HIF-1α and VHL proteins by Western blot. (C) miR-20b inhibitor or miR-20b did not affect the mRNA levels of HIF-1α. The cells of above (A) and (B) were used for real time RT-PCR to detect HIF-1α mRNA expression. (D) miR-20b targeted 3′-UTR of HIF-1α mRNA. The specificity of miR-20b to 3′-UTR of HIF-1α mRNA was identified as described in *Methods*. *, *P*<0.01, compared with HIF-1α 3′-UTR group.

To verify that HIF-1α is targeted by miR-20b, a blocking strategy was adapted here by introducing miR-20b inhibitor to normoxic H22 tumor cells, which indeed increased the protein level of HIF-1α (Figure 2A). This was not due to the more degradation of HIF-1α, since miR-20b inhibitor did not affect the VHL protein level (Figure 2A), which binds and degrades HIF-1α. On the other hand, the increase of miR-20b level of hypoxic tumor cells by transfecting miR-20b effectively decreased HIF-1α protein level (Figure 2B). Similarly, The VHL protein level was not altered (Figure 2B). In both cases, neither miR-20b inhibitor nor miR-20b affected the mRNA of HIF-1α (Figure 2C), suggesting that HIF-1α is a target for miR-20b. To confirm the direct interaction, a reporter vector system was used here. Transfection of HIF-1α 3′-UTR-containing luciferase reporter vector into CHO cells resulted in the decent fluorescence production. However, the cotransfection of miR-20b and reporter vector resulted in a significant decrease of luciferase activity (Figure 2D). Thus, miR-20b directly targets 3′-UTR of HIF-1α.

### miR-20b is downregulated by *HIF-1α*

**Figure 3:**
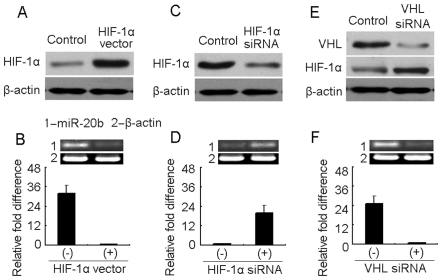
miR-20b is downregulated by HIF-1α. (A and B) HIF-1α decreased miR-20b expression in normoxic H22 cells. HIF-1α vector or mock vector was transfected into normoxic H22 cells. 72 hr later, the cells were used for HIF-1α analysis by Western blot (A) and miR-20b analysis by RT-PCR and real time RT-PCR (B). (C and D) HIF-1α siRNA was transfected into hypoxic H22 cells. 72 hr later, the cells were used for HIF-1α analysis by Western blot (C) and miR-20b analysis by RT-PCR and real time RT-PCR (D). (E and F) VHL siRNA was transfected into normoxic H22 cells. 72 hr later, the cells were used for VHL and HIF-1α analysis by Western blot (E) and miR-20b analysis by RT-PCR and real time RT-PCR (F).

The differential expression of miR-20b between normoxia and hypoxia raised the question of how miR-20b was downregulated in hypoxic tumor cells. To explore the underlying mechanism, we hypothesized that miR-20b was regulated by HIF-1. To test this, HIF-1α was forcedly overexpressed in H22 tumor cells by the transfection of its expressing vector (Figure 3A). As a result, the forcedly expressed HIF-1α caused the decrease of miR-20b in normoxia (Figure 3B). Meanwhile, by a comparable approach, we silenced HIF-1α expression in hypoxic tumor cells using HIF-1α siRNA (Figure 3C). As expected, the decrease of HIF-1α resulted in the increase of miR-20b in hypoxic tumor cells (Figure 3D). In addition, we transfected VHL siRNA to normoxic tumor cells to increase the HIF-1α protein level (Figure 3E). Under such condition, the miR-20b level was also reduced (Figure 3F). Taken together, these data suggested that HIF-1α downregulates miR-20b expression in tumor cells.

### miR-20b regulating VEGF further tunes tumor cells to adapt to the alteration of oxygen concentration

**Figure 4:**
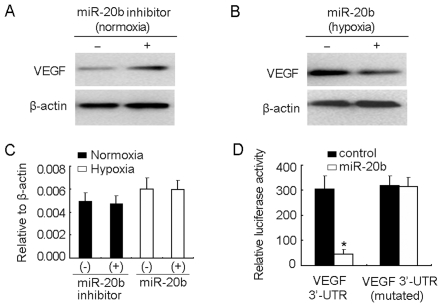
VEGF is targeted by miR-20b. (A) miR-20b inhibitor increased VEGF protein in normoxic H22 cells. miR-20b inhibitor or control oligonucleotide was transfected into normoxic H22 cells. 48 hr later, the cells were used to detected VEGF expression by Western blot. (B) miR-20b decreased VEGF protein in hypoxic H22 cells. miR-20b or control oligonucleotide was transfected into hypoxic H22 cells. 48 hr later, the cells were used to detect VEGF expression by Western blot. (C) miR-20b inhibitor or miR-20b did not affect the mRNA levels of VEGF. The cells of above (A) and (B) were used for real time RT-PCR to detect VEGF mRNA expression. (D) miR-20b targeted 3′-UTR of VEGF mRNA. The specificity of miR-20b to 3′-UTR of VEGF mRNA was identified as described in *Methods*. *, *P*<0.01, compared with VEGF 3′-UTR group.

Previous study has reported that miR-20b is a putative regulator of VEGF [17], the pivotal angiogenic factor in response to hypoxia. Here, we further hypothesized that VEGF was targeted by miR-20b thus to additionally tune tumor cells to adapt to the alteration of oxygen concentration. To test this, we performed the *in vitro* assay as described above (Figure 2). Under normoxic condition, the decrease the activity of miR-20b by transfecting miR-20b inhibitor resulted in the upregulation of VEGF protein (Figure 4A); under hypoxic condition, the increase miR-20b level by transfecting miR-20b however downregulated VEGF protein (Figure 4B). Nevertheless, the mRNA level of VEGF was not affected by either miR-20b inhibitor or miR-20b (Figure 4C). Moreover, cotransfection of miR-20b and VEGF 3′-UTR-containing luciferase reporter vector into CHO cells resulted in a significant decrease of fluorescence intensity (Figure 4D), suggesting that miR-20b may directly targets 3′-UTR of VEGF. Thus, the regulation of VEGF by miR-20b may further tune tumor cells to adapt to the alteration of oxygen concentration.

### Roles of miR-20b in tumor cells

**Figure 5:**
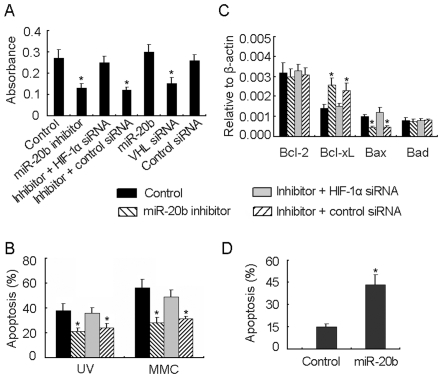
Differential expression of miR-20b affects different aspects of tumor cells. (A) miR-20b was required for H22 cell growth. H22 cells were transfected with miR-20b inhibitor or miR-20b inhibitor + HIF-1α siRNA or miR-20b or VHL siRNA or control oligonucleotide for 24 hr. Then the cells were seeded in 96-well plate (5×10^3^ per well) for 48 h in normoxia. The proliferation assay was performed with MTT Cell Proliferation Kit (Roche Diagnostics, IN) according to the manufacturer's instructions. *, *P*<0.05, compared with control. (B) Downregulation of miR-20b enhanced the resistance to apotosis. H22 cells were transfected with miR-20b inhibitor or miR-20b inhibitor + HIF-1α siRNA for 24 hr. Then the cells (1×10^6^) were irradiated by UVB (200 J/m^2^) or treated with mitomycin C (MMC, 10 µg/ml)for 12 h. The cells were stained with PE-Annexin V and 7-AAD for apoptotic analysis by flow cytometry. *, *P*<0.05, compared with control. (C) Analysis of the mRNA expressions of Bcl-2, Bcl-xL, Bax and Bad genes. H22 cells were transfected with miR-20b inhibitor or miR-20b inhibitor + HIF-1α siRNA for 72 hr. The cells were used for the analysis of gene expression by real time RT-PCR. *, *P*<0.05, compared with control. (D) Normoxic H22 cells were transfected with miR-20b for 24 hr. Then the cells were cultured under hypoxic condition for 12 h, and stained with PE-Annexin V and 7-AAD for apoptotic analysis by flow cytometry. *, *P*<0.05, compared with control.

miR-20b has been reported to accumulate in tumor cells and speculated to have an oncogenic role [18], [19]. Here, we further explored the biological significance of miR-20b in tumor cells based on the above study. Under normoxic condition, tumor cells have the capacity of vigorous proliferation and high expression of miR-20b, implying a possible role of miR-20b in tumor cell growth. To verify this, miR-20b inhibitor was transfected into normoxic H22 tumor cells to block the function of miR-20b. As a result, tumor cell growth was retarded significantly (Figure 5A). However, this retardation was rescued by the cotransfection of HIF-1α siRNA (Figure 5A), suggesting that miR-20b maintains tumor cell growth through its regulation of HIF-1α. In addition, the VHL knockdown also dampened H22 cell growth significantly (Figure 5A). Nevertheless, the further increase of miR-20b level in normoxic H22 tumor cells by the transfection of miR-20b seemed to accelerate tumor cell growth but not significantly (Figure 5A).

miR20b was downregulated in tumor cells in hypoxia. We then further asked whether the decrease of miR-20b contributed to a more malignant phenotype of cancer cells, such as the enhanced resistance to apoptosis, the feature of HIF-1α in tumor. To verify this, miR-20b inhibitor was transfected to normoxic H22 tumor cells. 24 hr later, the cells were treated with UV irradiation or chemodrug mytomycin C. The flow cytometric result showed that inhibition of miR-20b increased the apoptosis resistance of tumor cells, compared to the control (Figure 5B). Nevertheless, cotransfection of HIF-1α siRNA completely abolished the effect of miR-20b inhibitor (Figure 5B), suggesting that the decrease of miR-20b enhances the resistance to chemo- and radiotherapy through increasing the HIF-1α level. In line with these data, miR-20b inhibitor transfection could affect Bcl-2 family gene expression, including upregulation of Bcl-xL and downregulation of Bax in normoxic H22 tumor cells (Figure 5C). Finally, we tested the role of miR-20b in hypoxia. Normoxic H22 cells were transfected with miR-20b. 24 hr later, 1×10^6^ cells were cultured in hypoxia for 12 h for apoptosis detection by flow cytometry. The result showed that miR-20b transfection significantly increased the proportion of apoptotic cells, compared to control (Figure 5D). In addition, by combinatorial utilization of different algorithms, such as TargetScan, PicTar, and Sanger microRNA target, we searched all the candidate target genes for miR-20b. The result showed that Bcl-xL and Bax were not in the list of candidate genes, suggesting miR-20b indirectly regulating these genes. Taken together, these data suggested that HIF-1α-mediated downregulation of miR-20b is required for H22 cells to adapt the hypoxia.

## Discussion

The current concept on the adaptive responses to changes in tissue oxygenation is mainly formed from the study on pVHL-mediated degradation of HIF-1α. Here, we provide alternative mechanism that a single microRNA, miR-20b, by virtue of its differential expression in normoxic and hypoxic microenvironment, can finely tune the expressions of HIF-1α and VEGF in tumor cells, making them to adapt to different oxygen concentrations.

microRNAs are endogenous non-coding small RNAs of 19–23 nucleotides in length. The biological functions of microRNAs are involved in numerous cellular processes, including proliferation, differentiation, metabolism, and motility [20]–[22]. Coincidently, all these cellular processes can also be influenced by a transcription factor HIF-1 [23], [24], implying possible connection of HIF-1 and microRNAs. HIF-1 activity is controlled by the von Hippel-Lindau tumor suppressor protein (pVHL), a recognition component of the E3 ubiquitin ligase. Under normoxic condition, pVHL binds hydroxylated proline residues within HIF-1α and thereby targets HIF-1α to the 26S proteasomal degradation system (4–6). However, during hypoxia, proline hydroxylases are inactive and can not catalyze the hydroxylation of proline residues of HIF-1α, leading to the accumulation of HIF-1α in the cell. Besides this classical regulation machinery, in the present study, we additionally demonstrate that HIF-1α and VEGF is regulated by miR-20b in tumor cells. Intriguingly, miR-20b is also regulated by HIF-1α; VEGF can be transcriptionally activated by HIF-1 [25]. Such multiple regulation relationship among miR-20b, HIF-1α and VEGF therefore keep tumor cells to adapt different oxygen concentration for survival.

HIF-1α is a master regulator of oxygen homeostasis [4]. Unusually, its response to oxygen alteration happens at the protein level rather than mRNA level. The reason maybe is that the cells can immediately translate the preexisted mRNA into the protein so to adapt the hypoxia promptly. On the other hand, the degradation of HIF-1α protein through pVHL-mediated pathway is also extremely rapid with a half-life estimated to be less than 5 minutes [4]. Such regulation model for HIF-1α is efficient but too rigid in some physiological conditions. For instance, the formation of hypoxia in tissues such as embryo or tumor is a gradual process. Therefore, to reconcile this, a complementary mechanism may be required. Our findings of the reciprocal regulation of miR-20b and HIF-1α may be such a paradigm. Interestingly, VEGF is also targeted by miR-20b directly. Based on these findings, we propose that miR-20b cooperates with pVHL-mediated degradation pathway to effectively repress HIF-1α in normoxia. In order to adapt the gradual onset of hypoxia, inactivation of pVHL pathway is an initial response, which ceases the degradation of HIF-1α and leads to the increase of HIF-1α protein. However, to further increase of HIF-1α and VEGF proteins, tumor cells have to remove the inhibition of miR-20b on HIF-1α and VEGF by downregulating miR-20b. Such regulation model confers tumor cells to sense precisely the alteration of oxygen by the dynamic equilibrium between miR-20b and HIF-1α.

miR-20b has been reported to accumulate in tumor cells and might play an oncogenic role [18], [19]. Beyond consistence with these reports, our data here at least partially elucidate the mechanism underlying miR-20b-promoting tumorigenesis. We confirm the differential expression of miR-20b in different tumor regions in a mouse liver cancer model. Such differential expression of miR-20b may have different roles in tumor cells. High expression of miR-20b favors tumor cell growth in normoxia; low expression of miR-20b inhibits tumor cell growth but confers tumor cell more resistance to apoptosis in hypoxia. Although we show that the down-regulated miR-20b leads to an increase in the levels of the anti-apoptotic factor, BcL-xL and a decrease in the levels of the pro-apoptotic factor, Bax, the informatics analysis indicate that neither BcL-xL nor Bax as a candidate target gene for miR-20b. Considering the regulation of apoptosis genes by HIF-1α [26]–[29], the regulation of apoptosis genes by miR-20b may be ascribed to the reciprocal regulation of miR-20b and HIF-1α.

VEGF is one of the most potent stimulators of angiogenesis by stimulating the proliferation and migration of vascular endothelial cells [30]–[32]. In a rapidly growing tumor, the increased diffusion distances between the blood vessels and the oxygen-consuming cells [33], [34] lead to the decrease of oxygen delivery and local hypoxia, which trigger the upregulation of VEGF in an effort to ameliorate the hypoxic state. Regardless of the important role of VEGF in tumor hypoxia, our data show that miR-20b downregulates VEGF expression in tumor cells. Thus, the accumulation of miR-20b in tumor cells [18], [19] raises the question of how tumor cells adapt the hypoxia when the expression of VEGF is inhibited. A possible reason is that tumor stromal cells may act as the cellular source of VEGF. On the other hand, the downregulation of miR-20b may upregulate VEGF expression in tumor cells, which impairs the inhibitory effort on tumor cells by the decrease of miR-20b. Therefore, although our findings and others suggest that miR-20b may be a potential target in tumor therapy, the combination of targeting miR-20b and VEGF might be a better choice.

## Materials and Methods

### Ethics statement

All animal work was conducted according to relevant national and international guidelines. For details please refer to subsection entitled **Animals and cell lines**.

### Animals and cell lines

BALB/c mice, 6 to 8-week-old, were purchased from Center of Medical Experimental Animals of Hubei Province (Wuhan, China) for studies approved by the Animal Care and Use Committee of Tongji Medical College. Mouse tumor cell lines H22 (hepatocarcinoma), 4T1 (breast cancer), RM1 (prostate cancer), and B16 (melanoma) were purchased from the American Type Culture Collection (ATCC, Manassas VA) and China Center for Type Culture Collection (CCTCC, Wuhan, China), and cultured according to their guidelines. For hypoxic experiments, cells were incubated in a hypoxic incubator. The atmosphere was maintained at 1% O2, 5% CO2 in humidified environment at 37°C.

For tumor model, 2×10^5^ H22 tumor cells (BALB/c derived) in 50 µl 0.9% sodium chloride sterile saline solution were subcutaneously injected to the left flank of BALB/c mice. 20 days later, when the tumor size reached 9×9 (mm×mm), the marginal and central tumor tissues were cut with small surgical scissors for RT-PCR, Western blot or immunohistochemical analysis.

### Analysis of microRNAs by RT-PCR and quantitative RT-PCR

Total microRNAs were isolated from tumor cell lines using microRNA isolation kit (Ambion, Austin, TX). Reverse transcription primers for miR-199b, 18a, 20b and 155 were designed, respectively, with RNA mfold version 2.3 server (supporting information, Figure S4), and its specificity was identified according to our previous study [35]. 100 ng of enriched microRNA was used for the cDNA synthesis. A 67-bp cDNA product was amplified by PCR with primers: miR-199b, 5′-CTCACAGTAGTCTGCACA-3′ (sense); miR-18a, 5′-GGTAAGGT GCATCTAG TG-3′ (sense); miR-20b, 5′-CCCAAAGTGCTCATAGT G-3′ (sense); miR-155, 5′-CGGTTAATGCTAATTGTG-3′ (sense); common antisense primer, 5′-GACTGTTCCTC TCTTCCTC-3′.

For real-time PCR, the above primers and the Taqman probe [6-FAM]TTGCGACTAC ACACACACACACA[BHQ1a-6FAM] were mixed with TaqMan® Universal PCR Master Mix (Applied Biosystems, Foster City, CA). The reaction mixtures were incubated at 95°C for 10 min, followed by 40 cycles of 95°C for 15 s and 60°C for 1 min in Stratagene QRT-PCR instrument.

### Identification of the specificity of miR-20b to HIF-1α and VEGF mRNAs

A 450 bp or 240 bp fragment of 3′-UTR of HIF-1α or VEGF mRNA containing the target sequence (GCACTTT) of miR-20b was amplified by RT-PCR (HIF-1α, sense 5′-CTCTGAG CTCTATCTGGAAGGTATGTG -3′, antisense 5′-CCTCAAGCTTCAGTTAGTGTTAGACC C-3′; VEGF, sense 5′-TAACCATGTCACCACCACG-3′, antisense 5′- CCCAGAAACAACCC TAATC-3′). The fragment was designated as HIF-1α or VEGF 3′-UTR, and inserted into pMIR-REPORT™ luciferase reporter vector (Sac I and Hind III restriction enzyme sites; Ambion). Another expressing vector was also constructed by the insertion of a mutated HIF-1α or VEGF 3′-UTR in which the target sequence of miR-20b was mutated into GCAATTT using QuikChange® Site-Directed Mutagenesis Kit (Stratagene). Then, the recombinant reporter vectors with normal and mutated HIF-1α or VEGF 3′-UTR were cotransfected with miR-20b into CHO cells, respectively, using TransMessenger™ Transfection Reagent (Qiagen). The luciferase assay was performed according to the manufacturer's instructions.

### Transfection assay

The tranfection assay was performed as described in our previous study [35]. Briefly, miR-20b (Dharmacon, Lafayette, CO), miR-20b inhibitor (Ambion, Austin, TX), HIF-1α siRNA (Invitrogen, Carlsbad, CA), VHL siRNA (Invitrogen), and the corresponding control oligonucleotides were purchased. HIF-1α-expressing vector was constructed by inserting HIF-1α cDNA into pcDNA3.1 vector. For transient transfection, 200 pmoles of synthesized oligonucleotide or 2 µg of plasmid was mixed with 100 µl of Nucleofector solution (Amaxa, Gaithersburg, MD), and transfected into 3×10^6^ tumor cells by electroporation using Nucleofector 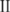 instrument. After transfection, the cells were allowed to recover by incubating for 4 h at 37°C, and then used for the following assays.

### Western blot

Cell lysates (30 µg of total protein) and prestained molecular weight markers were separated by SDS-PAGE followed by transfer onto nitrocellulose membranes. The membranes were blocked in TBST (Tris-buffered saline with 0.5% of Triton X-100) containing 5% nonfat milk, and probed with the indicated antibodies. After incubation with the secondary antibody conjugated with horseradish peroxidase, membranes were extensively washed, and the immunoreactivity was visualized by enhanced chemiluminescence according to the manufacturer's protocol (ECL kit, Santa Cruz, Santa Cruz, CA). All antibodies were purchased from Santa Cruze Biotechnology (Santa Cruz, CA).

### HIF-1α and VEGF detection by real time RT-PCR

The cDNA sequences of Hif1a, *Vegfa* and β-actin genes were retrieved from NCBI database. The primers were designed with the Oligo Primer Analysis 4.0 software. Real time RT-PCR was done as described previously [36]. The mRNA level of the detected gene was expressed as the relative level to that of β-actin.

### Immunohistochemistry

Central or marginal tumor tissues were surgically excised for the preparation of sections. The sections were fixed by acetone, and then incubated with 0.1% BSA to block the activity of endogenous peroxidase. Mouse-anti-human HIF-1α Ab (NB100-131SS, Novus Biologicals, Littleton, CO), biotinylated anti-mouse IgG (Santa Cruz), and streptavidin-conjugated horseradish peroxidase (Santa Cruz) were used for immunohistochemical staining.

### Proliferation assay

H22 cells were transfected with miR-20b inhibitor or miR-20b inhibitor+HIF-1α siRNA or miR-20b or VHL siRNA or control oligonucleotide for 24 hr. Then the cells were seeded in 96-well plate (5×10^3^ per well) for 48 h in normoxia. The proliferation assay was performed with MTT Cell Proliferation Kit (Roche Diagnostics, IN) according to the manufacturer's instructions.

### Apoptosis assay

H22 cells were transfected with miR-20b inhibitor or miR-20b inhibitor+HIF-1α siRNA for 24 hr. Then the cells (1×10^6^) were irradiated by UVB (200 J/m^2^) or treated with mitomycin C (MMC, 10 µg/ml)for 12 h. The apoptotic cells were analyzed with PE Annexin V Apoptosis Detection Kit (BD Biosciences, San Diego, CA) by flow cytometry.

### Statistics

Results were expressed as mean value ± SD and interpreted by Students't test. Differences were considered to be statistically significant when *P*<0.05.

## Supporting Information

Figure S1 The expressions of miR-18a, 155 and 199b are not affected by different oxygen concentrations. The H22 and B16 tumor cell lines were treated with different oxygen concentrations. The expressions of miR-18a, 155 and 199b were detected by real time RT-PCR. The expression level in the hypoxia groups was designated as 1.
(0.40 MB TIF)

Figure S2 The expressions of miR-20a, 106a, 18b, 92-2 and 363 are not affected by different oxygen concentrations. The H22 and B16 tumor cell lines were treated with different oxygen concentrations. The expressions of microRNAs were detected by real time RT-PCR. The expression level in the hypoxia groups was designated as 1.
(0.40 MB TIF)

Figure S3 HIF-1α is expressed in the central H22 tumor tissues but not in marginal tumor tissues. H22 tumor cells were subcutaneously injected to the left flank of BALB/c mice. When the tumor size reached 9×9 mm, central or marginal tumor tissues were surgically excised for the preparation of sections. The sections were used for immunohistochemical staining against HIF-1α. (A) The central tumor tissue was positively stained. (B) The marginal tumor tissue was negatively stained. (C) The interface of negative (marginal) and positive (central) staining of HIF-1α.
(2.19 MB TIF)

Figure S4 Stem-loop structure of reverse transcription primers for miR-199b, 18a, 20b and 155. The primers were designed with RNA mfold version 2.3 server. The 3′-end sequences of TAACCAA, CTATCTG, CTACCTG and ACCCCTA were complementary to the 3′-end sequence of miR-199b, 18a, 20b and 155, respectively.
(0.96 MB TIF)
