# Supplementary figures and images for: Regulation of HIF-1α and VEGF by miR-20b Tunes Tumor Cells to Adapt to the Alteration of Oxygen Concentration
Source: PLoS One. 2009 Oct 29;4(10):e7629. doi: 10.1371/journal.pone.0007629 (PMC2764090; doi:10.1371/journal.pone.0007629)

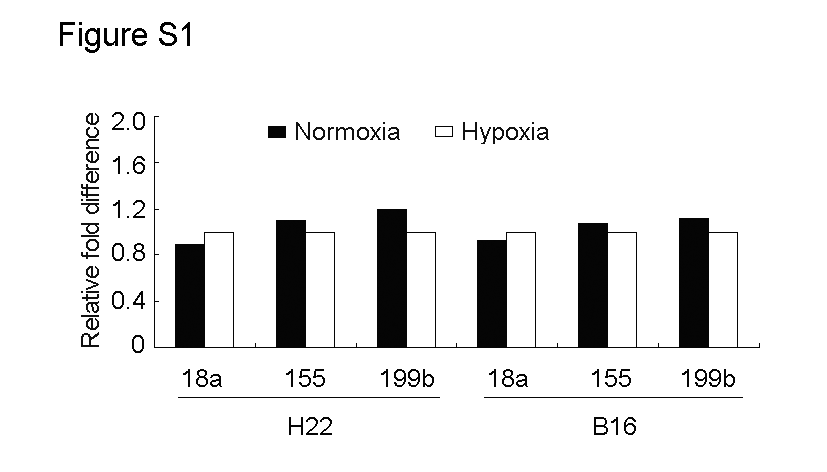

Supplement: Figure S1 — The expressions of miR-18a, 155 and 199b are not affected by different oxygen concentrations. The H22 and B16 tumor cell lines were treated with different oxygen concentrations. The expressions of miR-18a, 155 and 199b were detected by real time RT-PCR. The expression level in the hypoxia groups was designated as 1. (0.40 MB TIF) [file pone.0007629.s001.tif]

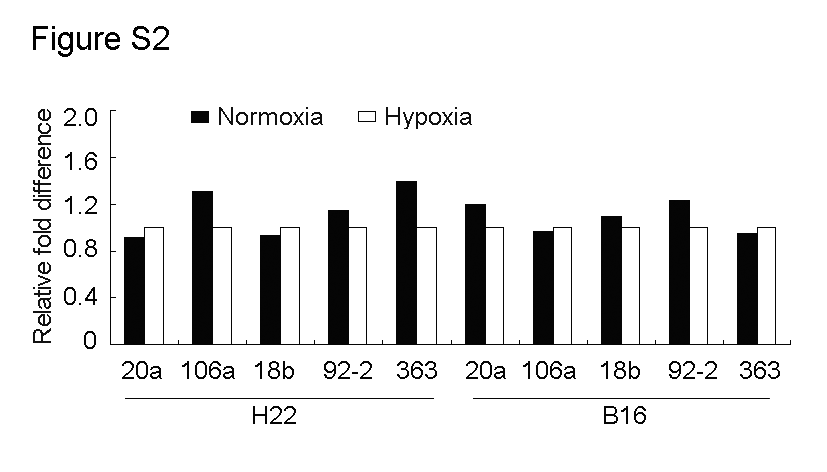

Supplement: Figure S2 — The expressions of miR-20a, 106a, 18b, 92-2 and 363 are not affected by different oxygen concentrations. The H22 and B16 tumor cell lines were treated with different oxygen concentrations. The expressions of microRNAs were detected by real time RT-PCR. The expression level in the hypoxia groups was designated as 1. (0.40 MB TIF) [file pone.0007629.s002.tif]

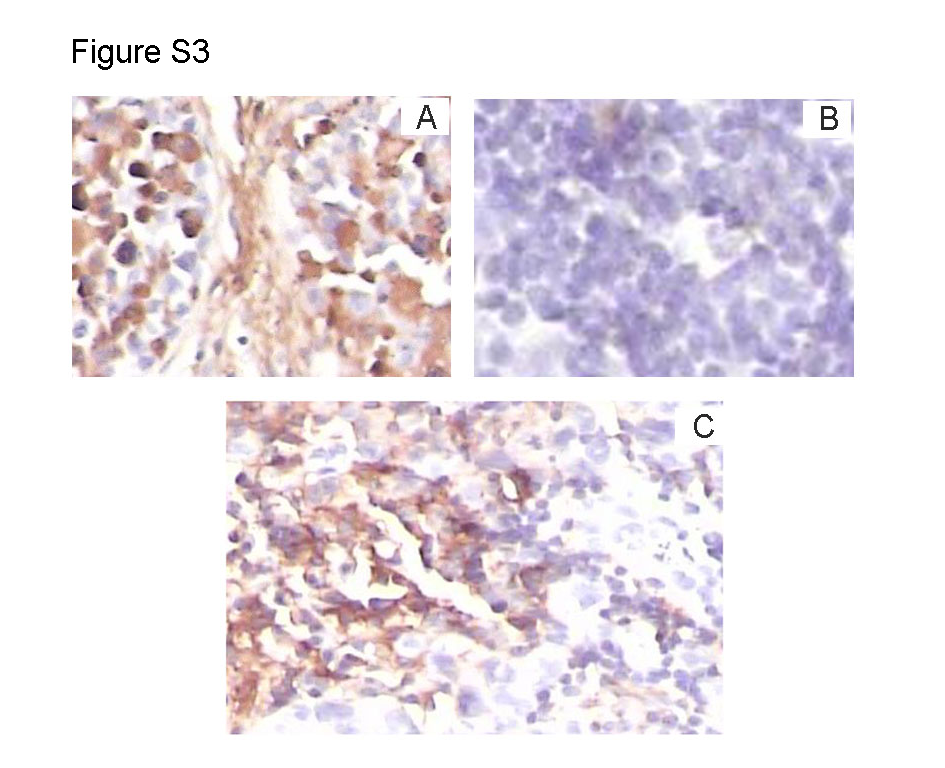

Supplement: Figure S3 — HIF-1α is expressed in the central H22 tumor tissues but not in marginal tumor tissues. H22 tumor cells were subcutaneously injected to the left flank of BALB/c mice. When the tumor size reached 9×9 mm, central or marginal tumor tissues were surgically excised for the preparation of sections. The sections were used for immunohistochemical staining against HIF-1α. (A) The central tumor tissue was positively stained. (B) The marginal tumor tissue was negatively stained. (C) The interface of negative (marginal) and positive (central) staining of HIF-1α. (2.19 MB TIF) [file pone.0007629.s003.tif]

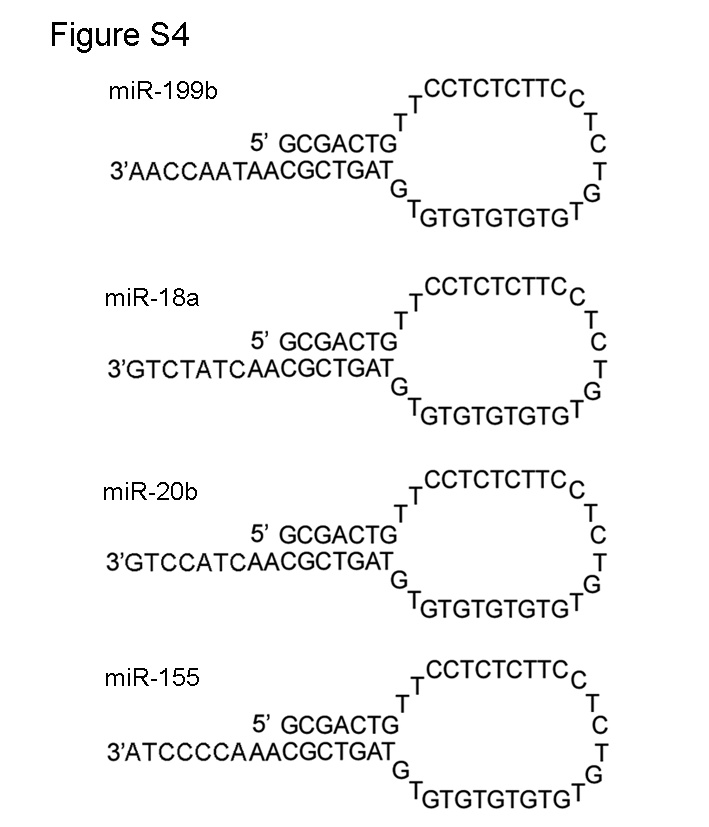

Supplement: Figure S4 — Stem-loop structure of reverse transcription primers for miR-199b, 18a, 20b and 155. The primers were designed with RNA mfold version 2.3 server. The 3′-end sequences of TAACCAA, CTATCTG, CTACCTG and ACCCCTA were complementary to the 3′-end sequence of miR-199b, 18a, 20b and 155, respectively. (0.96 MB TIF) [file pone.0007629.s004.tif]
